# Novel dimeric β-helical model of an ice nucleation protein with bridged active sites

**DOI:** 10.1186/1472-6807-11-36

**Published:** 2011-09-27

**Authors:** Christopher P Garnham, Robert L Campbell, Virginia K Walker, Peter L Davies

**Affiliations:** 1 Department of Biochemistry, Queen's University, Kingston, ON, K7L-3N6, Canada; 2 Department of Biology, Queen's University, Kingston, ON, K7L-3N6, Canada; 3 Department of Microbiology and Immunology, Queen's University, Kingston, ON, 7L-3N6, Canada

## Abstract

**Background:**

Ice nucleation proteins (INPs) allow water to freeze at high subzero temperatures. Due to their large size (>120 kDa), membrane association, and tendency to aggregate, an experimentally-determined tertiary structure of an INP has yet to be reported. How they function at the molecular level therefore remains unknown.

**Results:**

Here we have predicted a novel β-helical fold for the INP produced by the bacterium *Pseudomonas borealis*. The protein uses internal serine and glutamine ladders for stabilization and is predicted to dimerize via the burying of a solvent-exposed tyrosine ladder to make an intimate hydrophobic contact along the dimerization interface. The manner in which *Pb*INP dimerizes also allows for its multimerization, which could explain the aggregation-dependence of INP activity. Both sides of the *Pb*INP structure have tandem arrays of amino acids that can organize waters into the ice-like clathrate structures seen on antifreeze proteins.

**Conclusions:**

Dimerization dramatically increases the 'ice-active' surface area of the protein by doubling its width, increasing its length, and presenting identical ice-forming surfaces on both sides of the protein. We suggest that this allows sufficient anchored clathrate waters to align on the INP surface to nucleate freezing. As *Pb*INP is highly similar to all known bacterial INPs, we predict its fold and mechanism of action will apply to these other INPs.

## Background

Two extraordinary families of proteins have evolved to influence ice growth in opposite ways: antifreeze proteins (AFPs) that irreversibly adsorb to the surface of ice crystals to prevent their further growth [1]; and ice-nucleation proteins (INPs) that cause ice to form in solution at high sub-zero temperatures [2,3]. Whereas AFPs are small (Mr 3,000 - 35,000), and generally monomeric proteins, INPs are large (Mr >100,000) and function as multimers [4].

The tertiary structures of many AFPs are known, but none has been experimentally determined for an INP. Most INPs contain three distinct domains, with the majority of their mass residing within a highly repetitive central domain [5]. This domain consists of a variable number (ca. 50-80) of tandem 16-amino-acid (aa) repeats, with each repeat following the general consensus sequence of GYGSTxTAxxxSxLxA [6]. NMR and CD studies of synthetic bacterial INP peptides have not revealed a basic folding unit [6-9]. Molecular models of INP from the bacterium *Pseudomonas syringae *have included a planar array of anti-parallel β-strands [10] and a left-handed β-helix [6]. The latter was modelled on UDP-acetylglucosamine acyltransferease as an AFP-like β-helix with the β-stranded TxT motifs located within each 16-aa repeat aligning down one side of the protein and functioning as the site of ice nucleation. Interestingly, a 96-aa recombinantly-expressed fragment of *Ps*INP was shown to produce moderate levels of AFP activity [11], hinting that INPs and AFPs share a similar mechanism of action.

The Gram-negative bacterium *P. borealis *produces a 1244-aa INP (*Pb*INP) similar to all other known bacterial INPs [12]. Here we have predicted the structure of a 128-aa segment of the protein using a combination of homology-based modelling and molecular dynamics (MD) simulations. The right-handed β-helical model is stabilized by internal serine and glutamine ladders, and is predicted to dimerize via a highly conserved solvent-exposed tyrosine ladder. ach chain of the dimer contains two putative ice-nucleation sites, located opposite one another, and comprised of repetitive TQTA and SLTA β-strands. Each surface is flat and relatively hydrophobic, but also replete with hydrogen bond donors and acceptors; hallmarks of a typical AFP ice-binding site (IBS). MD simulations show each site is capable of ordering water molecules into an ice-like lattice, indicating that INPs use the same anchored clathrate water (ACW) mechanism of action that was recently elucidated for all AFPs [13]. Indeed, ACWs align across the entire width of both sides of the INP dimer, dramatically increasing the 'active' surface area of the protein and further strengthening the idea that size, and not fold or mechanism of action, is the primary discriminating factor between the two families of proteins with diametrically opposite functions regarding ice growth.

## Results and Discussion

### PbINP contains two ice-nucleating motifs per 16-aa repeat

**Figure 1:**
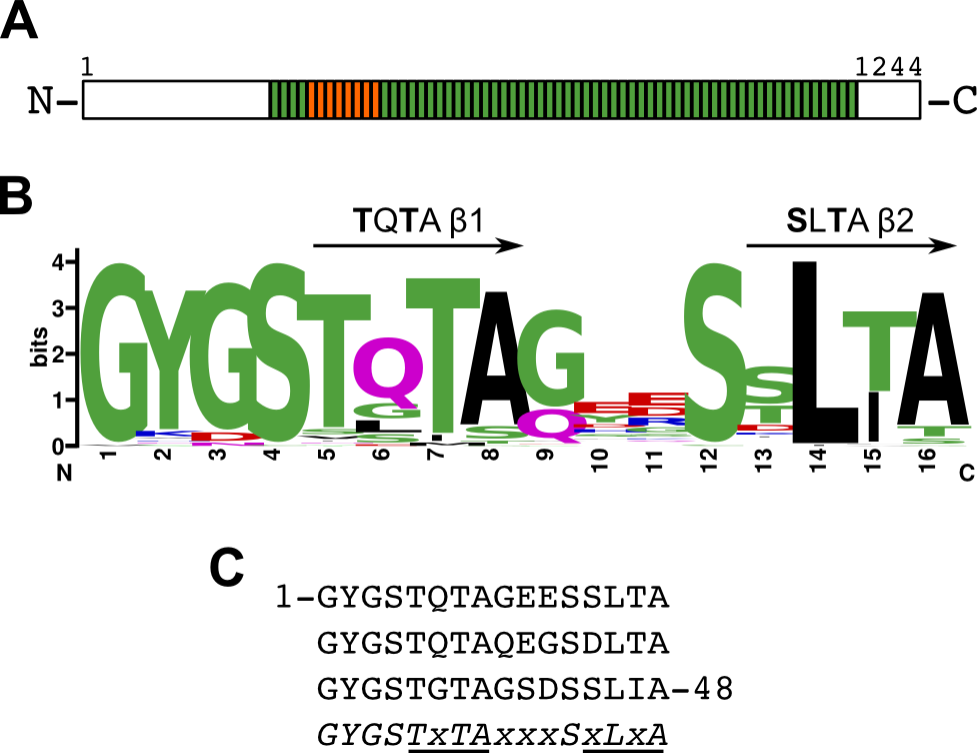
**Schematic representation of *Pb*INP and its repetitive amino acid sequence**. **A) **Each green or orange box represents one 16-aa repeat within the repetitive central domain. The non-repetitive N- and C-terminal domains are indicated by white rectangles. Boxes coloured orange are the eight repeats modelled in this study. **B) **WEBLOGO plot [41] of the 64 16-aa repeats of *Pb*INP. Hydrophobic residues are coloured black, acidic residues red, basic residues blue. Other residues are coloured green or purple. **C) **The amino acid sequence of *Pb*INP residues 217-265 is shown. The consensus amino acid sequence of the 3 × 16-aa block is indicated below in italics. Putative β-strands are underlined while x represents any amino acid.

**Figure 2:**
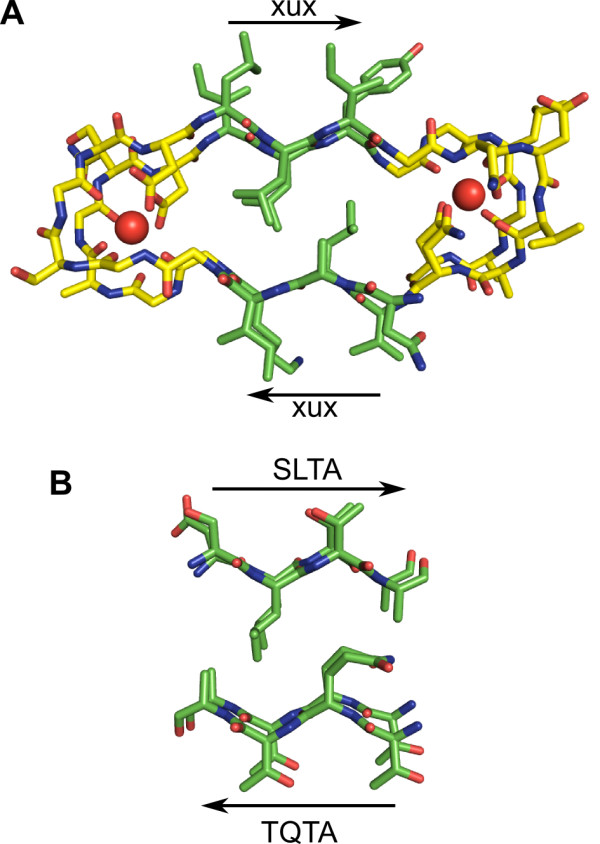
**Modelling of *Pb*INP**. **A) **The β-roll (residues 330-348) from alkaline protease (PDB 1KAP) was used as the starting template. Carbon atoms from the central β-strands of the structure are coloured green, while carbons from the Ca^2+^-binding turns are coloured yellow. Oxygen atoms are coloured red, nitrogen atoms blue, and Ca^2+ ^ions are shown as red spheres. Arrows indicate β-strands, with x representing any residue and u representing hydrophobic residues. **B) **The excised β-strands altered to conform to the sequence of *Pb*INP. The consensus sequence of each strand is indicated.

The highly repetitive central domain of *Pb*INP consists of 64 tandem 16-aa repeats (Figure 1A). Each repeat contains four distinct tetra-peptide motifs; two putative β-strands with consensus sequences of TQTA and SLTA, and two glycine- and serine-rich turns with consensus sequences of xxxS and GYGS (Figure 1B). Each 16-aa repeat forms part of a higher order 48-aa repeat, consisting of three tandem 16-aa sequences (Figure 1C). The tetra-peptide **T**Q**T**A and L**T**A β-strands (predicted solvent-exposed residues in bold) are highly similar to the **T**x**T **motifs that define the ice-binding sites (IBS) of β-helical insect and grass AFPs [14-17]. Previous modelling of the homologous *P. syringae *INP (*Ps*INP) based on UDP-acetylglucosamine acyltransferase (PDB 1LXA) predicted each 16-aa repeat of the protein formed one loop of a left-handed β-helix [6]. This aligned the **T**Q**T**A motifs down one side of he protein, forming a flat β-sheet that functioned as the site of ice nucleation. Since the 16-aa repeats of *Pb*INP (and all sequenced bacterial INPs) contain two putative **T**x**T**-like β-strands separated by glycine- and serine-rich tetra-peptide turns, each strand of *Pb*INP was predicted to align on opposite sides of a β-helical structure, forming two parallel β-sheets. For this reason, the β-roll from alkaline protease (PDB 1KAP) was chosen here as a starting template from which to model *Pb*INP. Located between its Ca^2+^-binding turns are two parallel β-sheets packed against one another (Figure 2A). The model of *Pb*INP was built by excising this central portion of the β-roll from alkaline protease and changing its residues to the appropriate **T**Q**T**A and **S**L**T**A motifs (Figure 2B). The xxxS and GYGS loops were manually built to connect the strands (see Additional file 1, Figure S1A), and the newly built 16-aa loop was duplicated, substituted with the appropriate *Pb*INP sequence, and aligned to the ensuing β-strands of the structure. This was performed until an eight-loop parallel β-helix was built that corresponds to residues 217-345 of *b*INP (see Additional file 1, Figure S1B).

### PbINP folds as a parallel β-helix

*Pb*INP was modelled as a right-handed β-helix due to the right-handedness of the modelling template. *Mp*AFP_RIV was previously modelled as a right-handed β-helix using the same β-roll template from alkaline protease and proved to be right-handed based on its crystal structure [13,18]. However, *Pb*INP could just as likely be left-handed, and the handedness of an ice-binding protein does not affect its ability to bind ice. Both L- and D-enantiomers of type I fish AFP and snowflea AFP display identical levels of antifreeze activity [19,20], while the non-homologous β-helical insect AFPs produced by *Tenebrio molitor *[15] and *Choristoneura fumiferana *[14] are right- and left-handed respectively, yet display near identical ice-binding sites and activity levels. Therefore, while we modelled *Pb*INP as a right-handed β-helix, we think that either a left- or right-handed version of the protein would be equally capable of efficiently nucleating ice.

**Figure 3:**
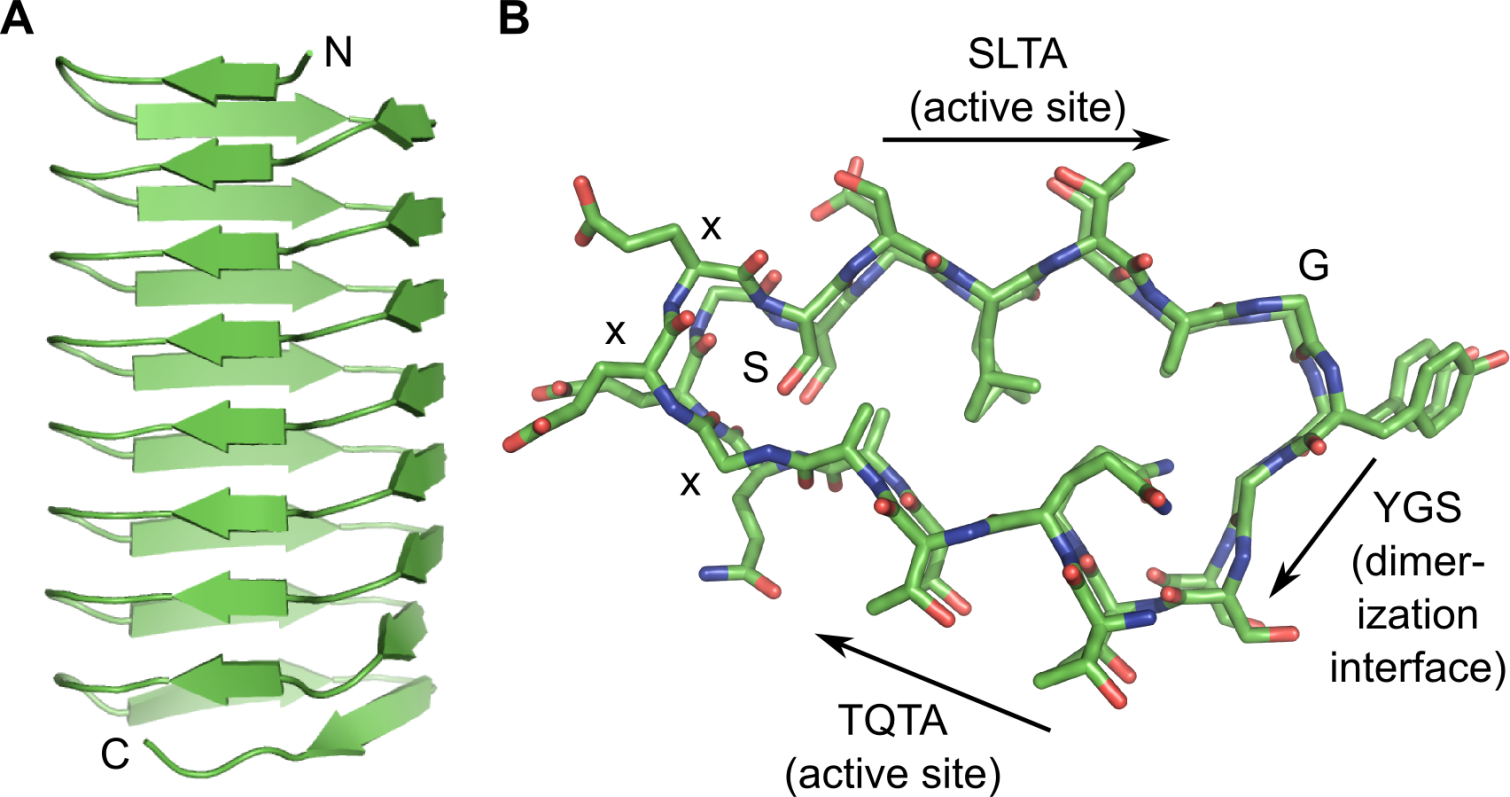
**β-helical model of *Pb*INP**. **A) **Cartoon representation of the energy-minimized average structure of *Pb*INP (residues 217-345) following a 5-ns MD simulation. β-strands are indicated by green arrows. The N and C termini are indicated. **B) **Cross-sectional view of *Pb*INP. The β-standed **S**L**T**A and **T**Q**T**A active sites are indicated, as is the β-stranded YGS multimerization interface. Oxygen atoms are coloured red, nitrogen atoms blue, and carbon atoms green.

To test the stability of the initial *Pb*INP model, a solvated 5-ns MD simulation was performed. The model stabilized at the 2-ns mark of the simulation and remained remarkably constant throughout the remainder of the trajectory (see Additional file 1, Figure S2A). An energy-minimized average structure of the model was calculated from the simulation's final 3 ns. The β-helix is roughly 40 Å in length × 20 Å wide × 5-10 Å thick (Figure 3A,B). A Ramachandran plot of the structure shows that all residues fall within allowable regions (see Additional file 1, Figure S2B). The protein contains a modest hydrophobic core, consisting of the alanine methyl groups at the end of each β-strand (TQTA and SLTA), the leucine side chain from the SLTA β-strands, and the side-chain methylene groups of the inward-projecting glutamine and serine residues from the TQTA and xxxS motifs respectively (Figure 3B). The leucine residues interdigitate with the side-chain methylene groups of the glutamine residues, further increasing stabilization. The polar groups of the internal glutamine and serine residues point toward the turns of the protein and away from the hydrophobic core, and main-chain inter-loop hydrogen bonds shield the hydrophobic core from the solvent.

**Figure 4:**
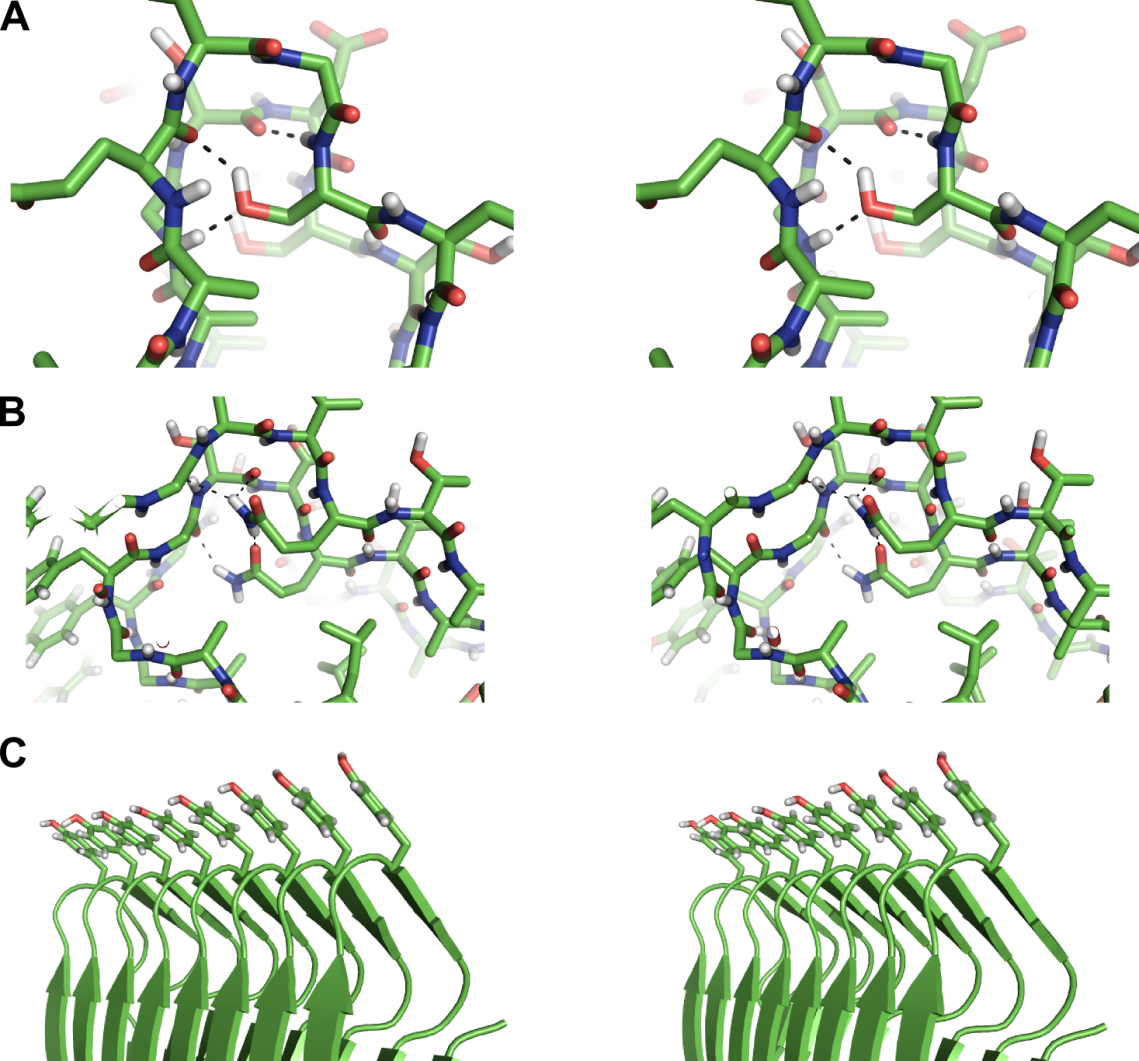
**The serine, glutamine, and tyrosine ladders of *Pb*INP**. **A) **Stereo view of the internal serine ladder. The colouring scheme is the same as in Figure 3. Hydrogen bonds are represented by black dashed lines. Hydrogen atoms are coloured white. Each serine -OH group forms two hydrogen bonds; one to the main-chain carbonyl oxygen from the first x residue of the xxxS motif of the same loop, and one to the main-chain amide nitrogen of the first x residue of the xxxS motif of the ensuing loop. The main-chain amide of each serine residue also hydrogen bonds to the main-chain carbonyl oxygen of the second x residue of the xxxS motif of the ensuing loop, further stabilizing the turn. **B) **Stereo view of the internal glutamine ladder. The side-chain amide of the glutamine forms hydrogen bonds with a side-chain carbonyl oxygen on the lower part of the loop, as well as with the main-chain carbonyl oxygen of the second glycine of the GYGS motif of the same loop and the serine of the GYGS motif of the ensuing loop. **C) **Stereo view of the external tyrosine ladder. The main chain of the protein is shown in cartoon format.

The inward-pointing serine and glutamine residues each form a ladder in opposite corners of the structure. The serines of the ladder reside in the final position of the xxxS motif that connects the TQTA β-strand to the SLTA β-strand (Figure 3B). Each Ser hydroxyl group hydrogen bonds to main-chain carbonyl oxygens and amide nitrogens of the same and ensuing xxxS motifs of the structure, while the main-chain amide nitrogen of each serine hydrogen bonds with the main-chain carbonyl oxygen of the second x residue of the xxxS motif of the ensuing loop (Figure 4A). The three x residues of the xxxS motif adopt an xbl β-arcade as defined by Hennetin *et al. *[21]. β-arcades of this configuration are typically followed by residues with short polar side chains that point inwards, allowing them to form stablizing H-bonds with the protein's main chain. This is clearly occurring in *Pb*INP with the highly conserved inward-pointing row of Ser residues. Also, the hyperactive β-helical AFP from the beetle *Tenebrio molitor *(*Tm*AFP) uses an internal serine ladder to stabilize turns within its structure [15].

The glutamine ladder is formed by the inward-pointing Gln residue of each TQTA motif (Figure 3B). Glutamines typically repeat two out of every three loops at this position, with the third loop usually having a glycine substitution. Glutamine side chains form hydrogen bonds with main-chain carbonyl oxygens and amide nitrogens of the turn immediately preceding the TQTA motif, as shown in stereo in Figure 4B. The void produced by the regularly-spaced glycine substitutions is filled by an internal water molecule that bridges adjacent glutamines and also forms hydrogen bonds to the main chain of the protein (see Additional file 1, Figure S3). The hyperactive β-helical *Tm*AFP uses internal water molecules to stabilize its structure [15]. Here it should be noted that these proteins have evolved to function at sub-zero temperatures where hydrogen bonding is relatively strong. While no structure in the Protein Data Bank contains a glutamine ladder, several β-helical proteins use very similar asparagine ladders to stabilize their folds [22], and TibA, a 104-kDa bacterial glycoprotein with both adhesin and invasin properties, is also predicted to contain an internal glutamine ladder [23].

### PbINP dimerizes via a solvent-exposed tyrosine ladder

The tightly packed core of the protein left no room for the tyrosine residue of the GYGS motifs, and as such, they were forced to adopt a solvent-exposed orientation during initial model building. During the course of the MD simulation, the tyrosine residues stacked on top of one another (Figure 3B, Figure 4C), and each tri-peptide YGS motif adopted a β-stranded conformation. Stacking of solvent-exposed aromatic residues has been observed within various proteins, and they typically reside in areas involved in receptor binding and/or dimerization. For example, InIJ, a β-helical leucine-rich repeat produced by the bacterium *Listeria monocytogenes*, contains multiple solvent-exposed and stacked aromatic residues within its predicted receptor recognition domain [24]. Most interestingly, engineered tyrosine ladders have been shown to promote the flatness [25] and dimerization [26] of OspA, a single-layer-β-sheet containing protein from the bacterium *Borrelia burgdorferi*. These results hinted at the possibility that *Pb*INP's tyrosine ladder might play a role in dimerization.

**Figure 5:**
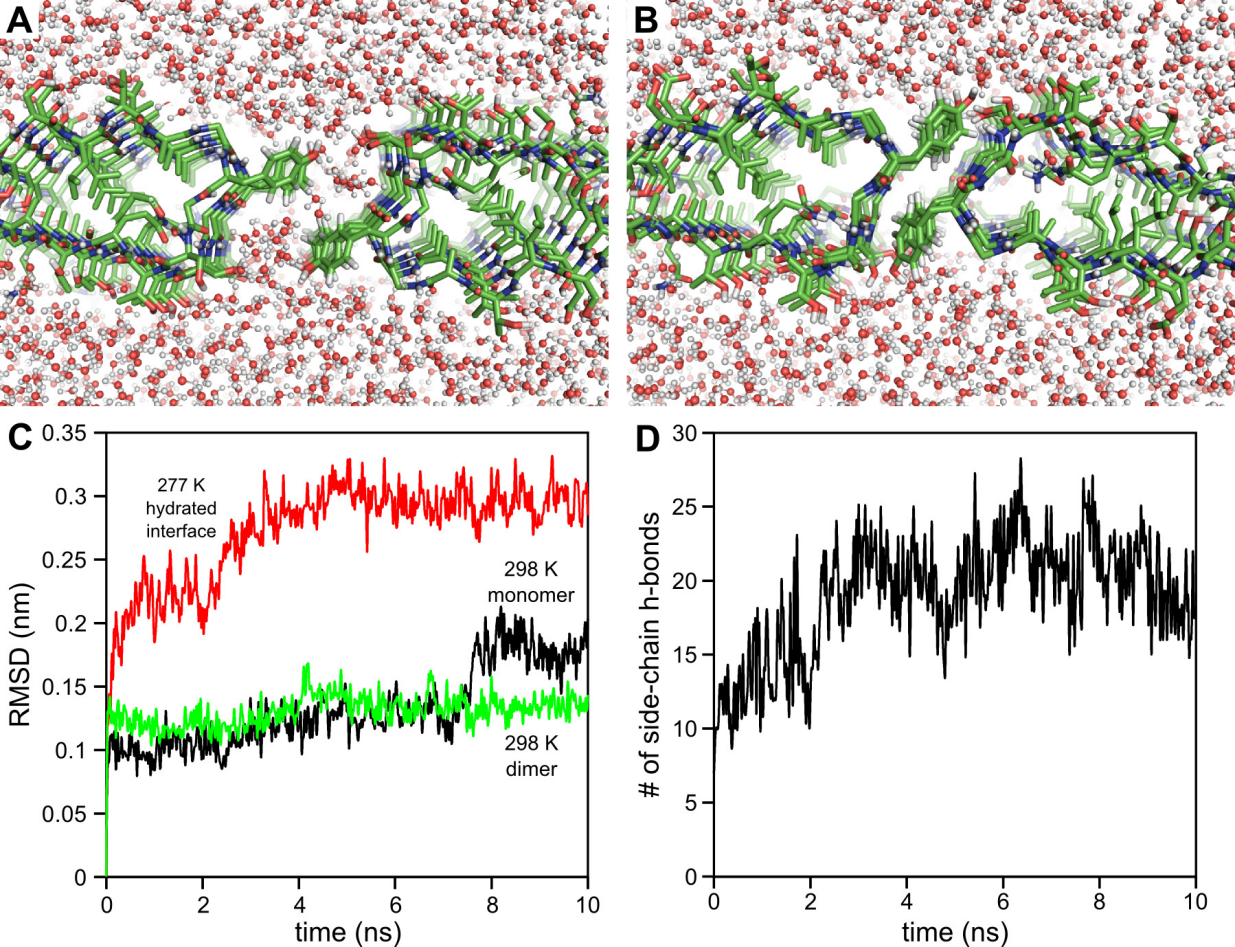
**MD simulations of the *Pb*INP dimer**. **A) **Two *Pb*INP monomers were aligned via their tyrosine ladders prior to the start of the MD simulation. Waters allowed to equilibrate at the dimerization interface prior to the start of the simulation are shown as red and white spheres. **B) **Final scene from the 10-ns simulation showing exclusion of waters from the dimerization interface. **C) **The Cα RMSD (nm) of the *Pb*INP dimer was plotted as a function of time (ns). The plots are of either the dimer starting from the hydrated interface (red line) or the energy-minimized average dimer simulated at the elevated temperature of 298 K (green line). A plot of the Cα RMSD (nm) of the *Pb*INP monomer (black line) run at the elevated temperature of 298 K was also plotted as a function of time (ns). **D) **The number of side-chain hydrogen bonds formed during the simulation was plotted as a function of time (ns).

**Figure 6:**
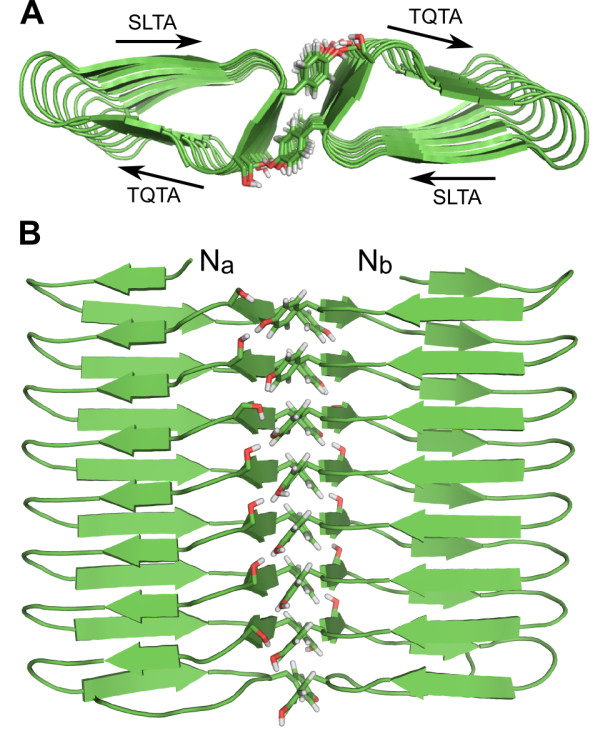
**Dimerization interface of *Pb*INP**. **A) **Cross-section end-on view of the energy-minimized average *Pb*INP dimeric structure. The protein is shown in cartoon format, with the tyrosine and serine residues that constitute the interface shown in stick format. The SLTA and TQTA active sites of each chain are indicated. The colouring scheme is the same as in Figure 2. **B) **Top-down view of the *Pb*INP dimer. The N termini of the a and b chains that constitute the dimer are indicated.

To test this idea, a model dimer of *Pb*INP was built by aligning two identical chains of *Pb*INP in a parallel manner such that their tyrosine ladders were oriented towards one another and their N termini were at the same end of the structure. This placed the SLTA surface of one chain on the same side of the structure as the TQTA surface from the opposite chain. A 10-ns MD simulation was then performed where water molecules were allowed to equilibrate at the dimerization interface prior to the start of the trajectory (Figure 5A). Immediately following the start of the simulation, the two chains moved towards one another, driven by the desolvation of the aromatic rings of the tyrosine residues (Figure 5B). The dimeric structure stabilized at the 5-ns mark of the trajectory and remained completely stable thereafter (Figure 5C). The energy-minimized average dimeric structure is shown in Figure 6. All waters were excluded from the dimerization interface as the aromatic rings of each tyrosine residue packed tightly against the β-stranded glycine residues of the opposite chain's GYGS motif (Figure 5B). Interestingly, the hydroxyl group of each tyrosine formed part of an intercalated hydrogen-bond network with the side-chain hydroxyl groups of the outward-projecting serine residues of the opposite chain's GYGS motifs (Figure 6). This is exemplified by the number of side-chain hydrogen bonds that formed during the simulation, increasing from 7 at the start to an average of 20 upon stabilization of the dimerization interface (Figure 5D).

To test the stability of the dimer, 10-ns MD simulations were performed on the energy-minimized average dimeric structure at the elevated temperatures of 298 K and 310 K (Figure 5C, Additional file 1 - Figure S4). In each case, the dimer remained stable throughout the trajectory, with no dissociation of individual chains, and complete exclusion of water molecules at the dimerization interface. Identical MD simulations were performed on the *Pb*INP monomer, and in each case, a partial unravelling of the C terminus of the protein occurred (at the 8-ns mark of the 298 K simulation and the 4-ns mark of the 310 K simulation) (Figure 5C, see Additional file 1 - Figure S4). These results demonstrate that dimerization increases the stability of *Pb*INP.

**Figure 7:**
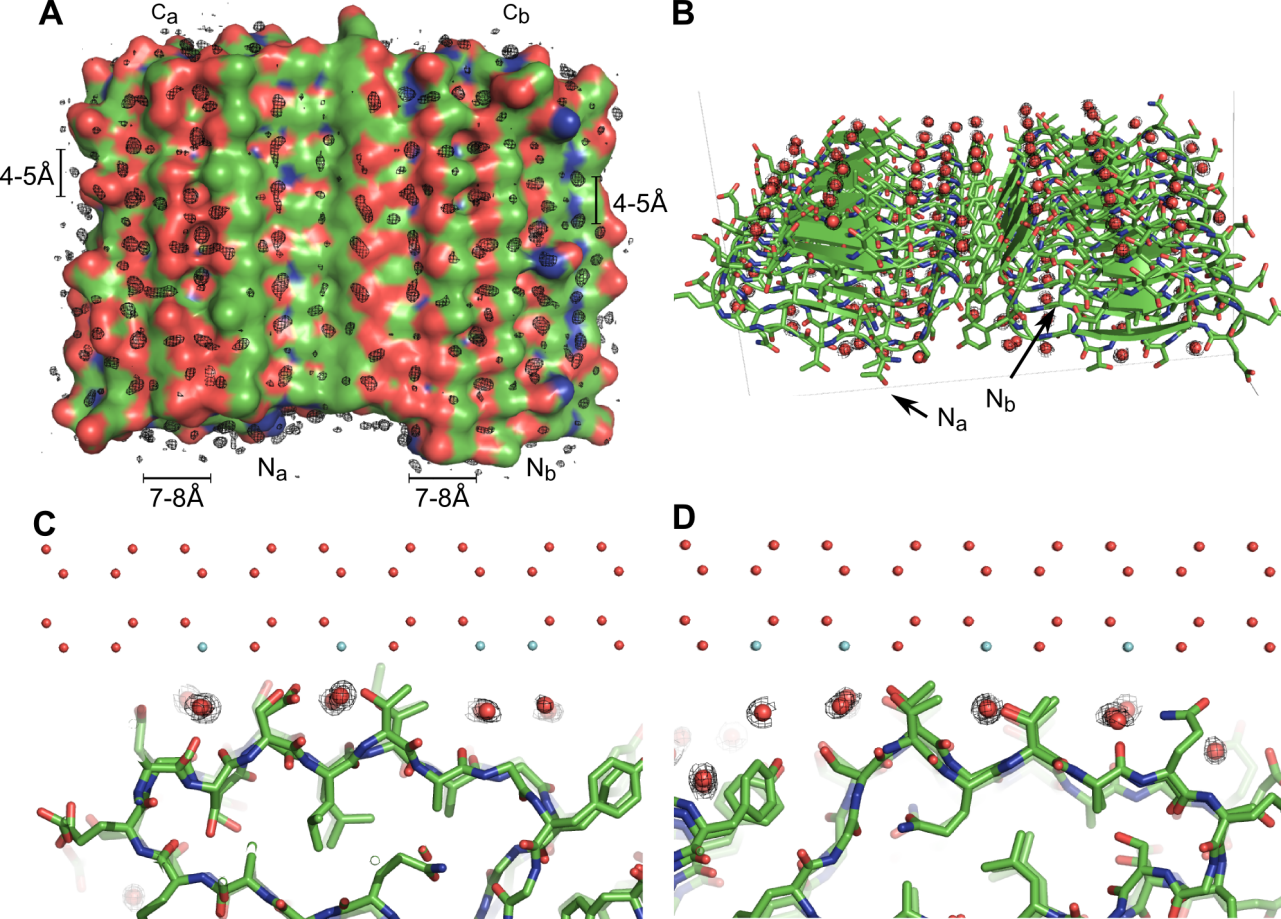
**Anchored clathrate waters on the ice-nucleating sites of *Pb*INP**. **A) ***Pb*INP is shown in surface mode, with oxygen atoms coloured red, nitrogen atoms blue, and carbon atoms green. Water electron density is shown as black mesh contoured at σ = 6. The N and C termini of each chain of the dimer are indicated. **B) **Anchored clathrate waters (red spheres) built into the electron density (contoured at σ = 6) align across the entire surface of *Pb*INP. **C) **Match of anchored clathrate waters present down the SLTA side of *Pb*INP to the primary prism plane of ice. Ice lattice waters are indicated by small red spheres, while those coloured aqua match the positions of waters on the ice nucleating site of the protein. **D) **Same as in C), except highlighting the match of anchored clathrate waters aligned down the TQTA site of the *Pb*INP dimer to the primary prism plane of ice.

We also attempted to model *Pb*INP as an anti-parallel dimer (see Additional file 1, Figure S5). The model remained intact following a 10-ns solvated MD simulation, however, a twist developed in the structure that did not develop in the parallel dimer (Figures 6, 7). In an anti-parallel orientation, the hydroxyl groups of the solvent-exposed rank of serine residues from one chain are unable to form an intercalated hydrogen-bond network with the hydroxyl groups of the tyrosine residues of the opposite chain. This lack of interaction allowed the twist to occur and therefore spoiled the flatness of the dimer. Flatness is a defining characteristic of ice-binding proteins, and as such, we think it is unlikely that *Pb*INP would dimerize in an anti-parallel manner.

### The ice nucleating surfaces of PbINP are highly conserved

*A*s previously mentioned, when *Pb*INP is modelled as a parallel dimer, the SLTA surface of one chain resides on the same side of the dimer as the TQTA surface of the other chain (Figure 6A). This creates a flat surface on both sides of the dimer, each spanning the entire width of the structure. The amino acid composition of both the TQTA and SLTA surfaces is highly conserved, as is the area in between them created by the dimerization interface. More specifically, the solvent-exposed first and third position of the **T**Q**T**A motifs have threonines present at these positions 87% and 92% of the time respectively. The solvent-exposed first and third position of the **S**L**T**A motifs show greater variation, however, serines and threonines are present at the first position 71% of the time (48% serine, 23% threonine), while threonine and isoleucine are present at the third position 98% of the time (60% threonine, 38% isoleucine). The rare substitutions at these positions are aspartate, valine and leucine. The dimerization interface is the most highly conserved portion of the whole protein, with the intercalated hydroxyl groups of the tyrosine and serine residues present 90% and 98% of the time, respectively.

### PbINP orders water molecules via the anchored clathrate water mechanism

This high degree of amino acid conservation endows the *Pb*INP dimer with two sides that are flat, and relatively hydrophobic, but also contain many hydrogen bond donors and acceptors. These characteristics define the IBS's of all AFPs, and as such, raise the possibility that INPs function by the ACW mechanism of AFP action [13]. The ACW mechanism states that the relative hydrophobicity of an AFP's IBS orders water molecules into an ice-like lattice, and this lattice is then anchored to the surface of the protein via hydrogen bonds.

To investigate the water ordering potential of *Pb*INP, a 2-ns MD simulation was performed on the energy-minimized average dimeric structure at a temperature of 273 K. The TIP5P water model was used as it accurately represents the behaviour of water during an MD simulation [27,28]. Waters within 10 Å of *Pb*INP's surface were extracted at 20-ps intervals throughout the course of the simulation and their crystallographic structure factors were calculated followed by a Fourier transform, giving the electron density of all extracted waters. Strong electron density with ice-like spacing was present across the entire width of the protein's surface (Figure 7A). Waters built into this density aligned down the troughs created by the outward projecting residues of the **T**Q**T**A and **S**L**T**A motifs, down the flat areas immediately preceding and following these motifs, and across the dimerization interface as well (Figure 7B). These waters closely match the 4.5 Å × 7.35 Å spacing of waters on the primary prism plane of ice, and as such, have revealed a potential orientation of a nascent ice crystal on the surface of the INP (Figures 7C,D).

**Figure 8:**
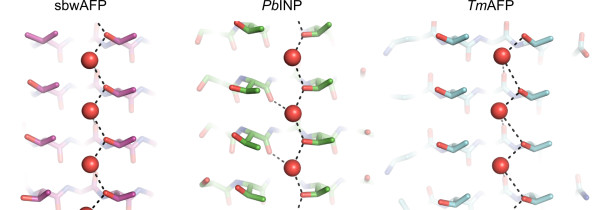
**Anchored clathrate water comparison**. Waters located down the TQTA trough of *Pb*INP (middle image) show near identical coordination as compared to the ice-like waters located down the TxT troughs of *Tm*AFP (PDB 1EZG (chain A)) (right image) and sbwAFP (PDB 1M8N (chain A)) (left image) as revealed by X-ray crystallography. Hydrogen bonds are represented by black hatched lines, while oxygens are coloured red, and nitrogens dark blue. Waters are represented as red spheres.

As a positive control, the same simulation was performed on *Mp*AFP_RIV, a hyperactive Ca^2+^-dependent β-helical AFP produced by the Antarctic bacterium *Marinomonas primoryensis*. *Mp*AFP_RIV was chosen as the positive control because the positions of the ACWs on its IBS are known [13]. The simulation accurately predicted the position of the ice-like waters on the IBS of the protein (see Additional file 1, Figure S6A,B). Indeed, aligning 22 manually-built waters to their crystallographic counterparts on the IBS of chain B of *Mp*AFP_RIV produced an RMSD of only 0.51 Å. Further proof of the accuracy of the MD simulation is evidenced by the identical coordination of waters present down the trough created by the TQTA motifs of *Pb*INP as compared to the waters located down the IBS TxT troughs of the β-helical insect AFPs (*Tm*AFP and sbwAFP) as revealed by X-ray crystallography (Figure 8).

### Dimerization increases the active surface area of PbINP

The temperature at which an INP nucleates ice is dependent upon its oligomerization state [4]. Approximately one INP monomer is capable of nucleating ice at a temperature of -12°C, while an aggregate of at least 50 INP monomers is required for ice nucleation in the -2°C to -3°C temperature range. However, due to the lack of an experimentally-determined INP structure, the mechanism by which an INP oligomerizes has remained a mystery. Previously, there have been only two attempts to predict how an INP might oligomerize. Wu *et al. *[12] speculated that overlapping protein-protein interactions between the two flat IBS motifs could generate 'stairs' of INPs, facilitating ice growth along the discontinuities. Prior to that, however, Kajava and Lindow [10] modelled *Ps*INP as an array of interdigitating anti-parallel β-strands that formed a flat ice-nucleating array upon oligomerization. While intriguing, the model was created prior to the structural determination of several β-helical AFPs [13-15], and as such, it overlooked the potential water-organizing capabilities of the TQTA and SLTA motifs when aligned as parallel β-strands in a flat β-sheet. As previously mentioned, Graether and Jia [6] modelled an INP as a β-helix, but the potential for oligomerization was not discussed. In that model, the serine of the GYGS motif pointed inwards and was not solvent exposed as predicted in this study. This would prevent formation of the intercalated hydrogen-bond network between the serine and tyrosine residues of the GYGS motifs from opposing chains in the dimer. Nevertheless, both β-helical models predict a solvent-exposed orientation for the tyrosine residue of the GYGS motif, and this argues against INP peptide studies that suggest it points towards the interior of the structure [8,9]. It is likely that short peptides adopt conformations in solution that are not representative of the full-length protein.

**Figure 9:**
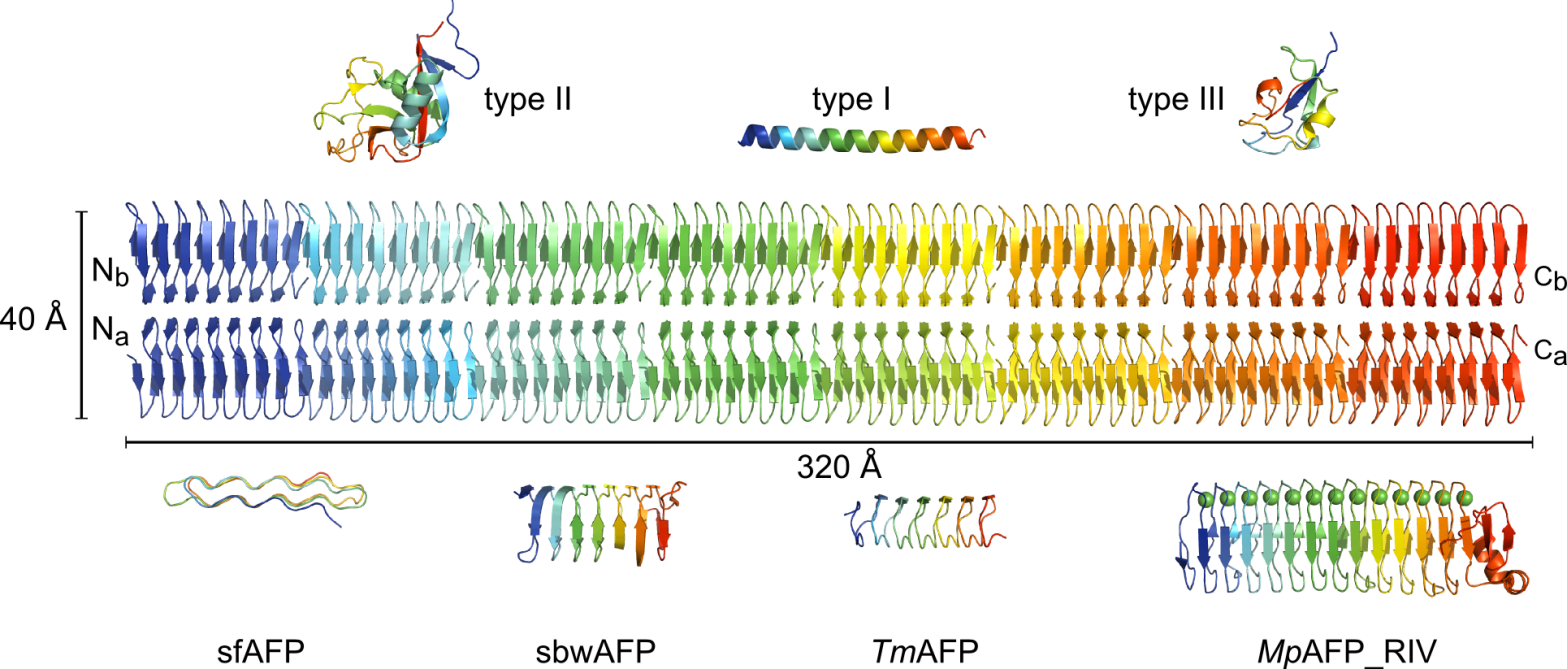
**Size comparison of *Pb*INP to other known AFPs**. Eight dimers of the *Pb*INP model were aligned end to end to approximate the size of the repetitive central domain of the protein (ca. 320 Å in length). The N and C termini are indicated. The crystal structures of all known AFPs are aligned with their IBSs facing *Pb*INP. Each protein is rainbow coloured with blue representing the N terminus and red representing the C terminus. All structures are to scale. Type I AFP (PDB 1WFA), type II AFP (PDB 2ZIB), type III AFP (PDB 1HG7), *Mp*AFP_RIV -*Marinomonas primoryensis *region IV AFP (PDB 3P4G), sbwAFP - spruce budworm AFP (PDB 1M8N), sfAFP - snow flea AFP (PDB 2PNE), *Tm*AFP - *Tenebrio molitor *AFP (PDB 1EZG).

**Figure 10:**
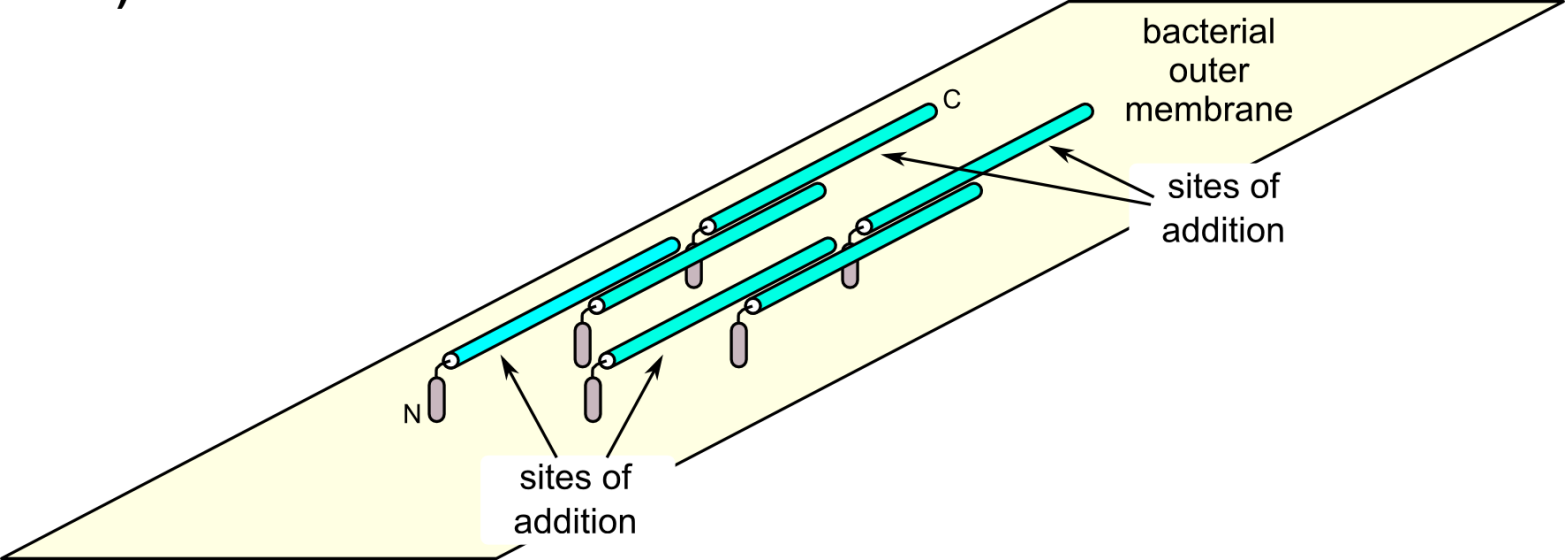
**Offset *Pb*INP dimerization about the surface of *P. borealis***. *Pb*INP monomers are shown as individual tubes, with the highly repetitive central domains coloured light blue and the non-repetitive N termini coloured grey. The N terminus of the first *Pb*INP chain of one multimer is indicated, as is the C terminus of the last *Pb*INP chain of the same multimer. To increase active surface area, individual *Pb*INP chains could add to the exposed sections near the two termini.

As a dimer with two bridged active sites on opposite sides of the structure, the potential ice-nucleating surface area of *Pb*INP is dramatically increased (Figure 9). A full-length *Pb*INP dimer would have an active surface area of ca. 25, 600 Å^2 ^(40 Å × 320 Å × 2), a value slightly larger than the 20, 100 Å^2 ^minimum surface area required for an ice embryo to continue growing at -12°C [29] (the temperature at which a single INP molecule is active [4]). By dimerizing in an offset manner, *Pb*INP monomers could form extended oligomeric structures that would increase the active surface area of the complex without occluding it, therefore raising the temperature at which ice nucleation occurs (Figure 10). In this scenario, the repetitive central domain of each *Pb*INP chain runs parallel to the surface of the bacterium, with the non-repetitive N terminus of each chain serving as a membrane anchor. Multiple arrays of offset dimers could increase the active *Pb*INP surface area on the outer membrane of the bacterium. Following initial construction of the monomeric model of *Pb*INP, it was thought the solvent-exposed tyrosine ladder might play a role in anchoring the protein to the membrane. However, several studies have shown the N-terminal domain alone is capable of performing this task [30,31], and it therefore would seem redundant to have other segments of the protein anchored to the bacterium, which would restrict the protein's access to solvent. Further investigation into the tertiary and quaternary structures of bacterial INPs, both *in vitro *and *in vivo*, are required and this oligomerization scenario is just one theoretical way of extending the ice nucleation surface.

Even without oligomerization, the size of *Pb*INP is significantly larger than any known AFP (Figure 9). This fact, combined with an INP's ability to order water molecules via the ACW mechanism, further strengthens the idea that size, and not a particular protein fold or mechanism of action, is the primary discriminating factor between the two families of proteins with diametrically opposite functions.

## Conclusions

This paper demonstrates through the use of homology modelling and MD simulations that bacterial INPs are able to fold as novel β-helical dimers. De-solvation of solvent-exposed tyrosine ladders drives INP dimerization, and this allows the ice nucleation sites of the protein to extend as a continuum across the width of the dimer. Both sides of the dimer order water molecules into an ice-like lattice using the anchored clathrate water mechanism of action. Offset dimerization can allow INP oligomerization without active site occlusion. This increases the active surface area of the INP, therefore raising the temperature at which ice nucleates.

## Methods

### Initial model building

All model building was performed using a combination of the programs PyMOL [32] and SYBYL (version 6.4, Tripos Associates, St. Louis, MO). PyMOL was used to excise the β-roll from alkaline protease (PDB 1KAP), remove the roll's Ca^2+^-binding turns, and change its tri-peptide xux β-strands (where x is any amino acid and u is a hydrophobic residue) to the appropriate TQTA and SLTA motifs of *Pb*INP. SYBYL was used to manually build and energetically minimize the xxxS and GYGS loops that connect the TQTA and SLTA β-strands. Finally, PyMOL was used to copy and expand the 16-aa loop until an 8-loop β-helix was completed (corresponding to residues 217-345 of *Pb*INP).

### Molecular Dynamics simulations of initial model

All MD simulations were performed using the program Gromacs v. 4.5.3 [33]. Prior to all full-scale MD simulations described below, energy minimization and a 50-ps position-restrained MD simulation was performed to relax the solvent around the protein. Berendsen temperature and pressure coupling were applied in all cases, and the GROMOS96 43a1 force field and SPC water model were used unless stated otherwise. The initial model of *Pb*INP (residues 217-345) was solvated in a box containing 6239 water molecules and 14 Na^+ ^ions to offset the charge of the protein. A full-scale 5-ns MD simulation was then performed at 277 K. The average structure of the final 3 ns of the simulation was calculated and energy minimized *in vacuo*. A Ramachandran plot of the protein was generated using the program PROCHECK [34].

### PbINP dimer construction and MD simulations

The dimer of *Pb*INP was built by duplicating the protein, rotating it 180° about the long axis of the structure, and then placing the duplicated chain's tyrosine ladder in close proximity to the tyrosine ladder of the original chain. This parallel arrangement of the helices placed their N termini at the same end of the dimer. The dimer was solvated in a box containing 13,401 waters and 28 Na^+ ^ions to offset the charge of the protein. The protein was subjected to a 10-ns full-scale MD simulation. An average structure of the protein from the final 5 ns of the simulation was calculated and energy minimized in the same manner as previously mentioned. This structure was then re-solvated and subjected to a 10-ns full-scale MD simulation at 298 K to test the stability of the dimeric protein. A single chain from the dimer was also subjected to the same simulation to test its stability at the elevated temperature of 298 K.

### PbINP dimer hydration studies

A 2-ns MD simulation was performed at the temperature of 273 K on the energy-minimized average dimeric structure of *Pb*INP to investigate the hydration of the protein. As a positive control, chain B from region IV of the AFP produced by the Antarctic bacterium *Marinomonas primoryensis *(*Mp*AFP_RIV) (PDB 3P4G) was subjected to the same simulation. The OPLS-aa force field [35,36] along with the TIP5P water model [27] were used in each case. To determine the probability density of water molecules, the following protocol was followed for each MD simulation. Coordinates of the system were extracted at 20-ps intervals throughout the course of the simulation (100 coordinate sets total/simulation). After superimposing these 100 structures by performing a least-squares fit on the protein Cα atoms, the waters within 10 Å of the protein were extracted and their electron densities were determined by calculating crystallographic structure factors followed by a Fourier transform using the programs SFall [37] and FFT [38] respectively of the CCP4 software suite [39]. The electron densities were calculated with the protein and water in a P1 unit cell equivalent to the box size used for the MD simulation. The FFT calculation used a grid spacing of 0.33 Å and a resolution of 1 Å. Waters were then manually built into the density using the program Coot [40].

## Abbreviations

aa: amino acid; ACW: anchored clathrate water; AFP: antifreeze protein; IBS: ice-binding site; INP: ice nucleation protein; MD: molecular dynamics; *Mp*AFP_RIV: *Marinomonas primoryensis *region IV antifreeze protein; *Pb*INP: *Pseudomonas borealis *ice nucleation protein; *Ps*INP: *Pseudomonas syringae *ice nucleation protein; sbwAFP: spruce budworm antifreeze protein; sfAFP - snow flea antifreeze protein; *Tm*AFP: *Tenebrio molitor *antifreeze protein.

## Authors' contributions

CPG performed all modelling and molecular dynamics simulations. The manuscript was written by CPG with editorial input from RLC, VKW, and PLD. All authors read approved the final manuscript.

## Supplementary Material

Additional file 1 **Supplementary Information**. Supplementary Figures 1,2,3,4,5,6. Click here for file
