# Supplementary material for: Novel dimeric β-helical model of an ice nucleation protein with bridged active sites
Source: BMC Struct Biol. 2011 Sep 27;11:36. doi: 10.1186/1472-6807-11-36 (PMC3196904; doi:10.1186/1472-6807-11-36)
Supplement: Additional file 1 — Supplementary Information. Supplementary Figures 1,2,3,4,5,6. [file 1472-6807-11-36-S1.PDF]

Supplementary Information

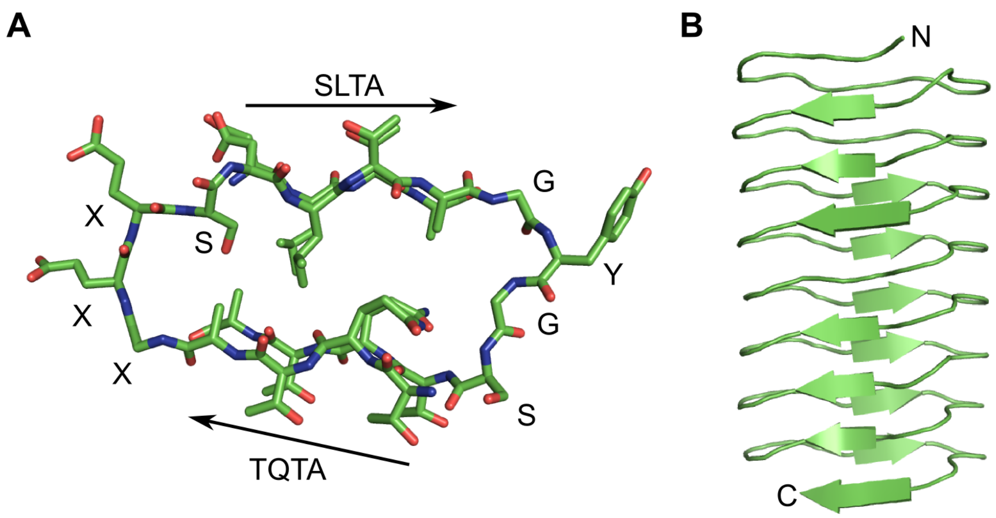

Figure S1 – Model of *Pb*INP prior to solvated MD simulation. A) The tetra-peptide xxxS and GYGS loops were manually built to connect the TQTA and SLTA β-strands of the altered β-roll from alkaline protease. **B)** The constructed 16-aa loop was duplicated, changed to the appropriate *Pb*INP residues, and then aligned to the β-strands of the ensuing loop. This was done eight times in total, producing a β-helical structure of *Pb*INP residues 217-345.

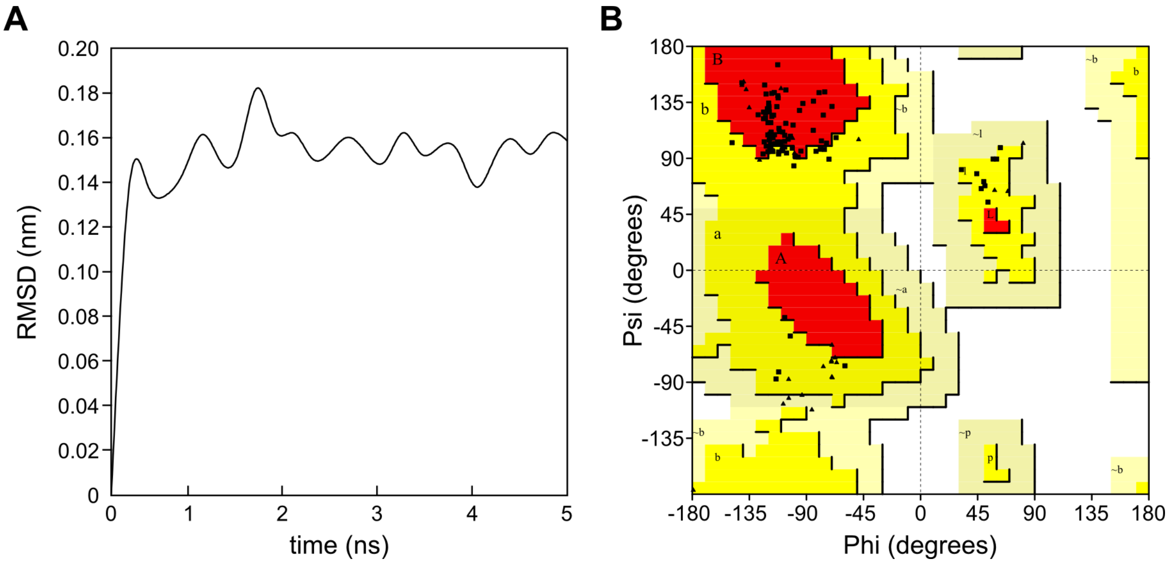

Figure S2 – RMSD plot of the 5-ns MD simulation and Ramachandran plot of averaged structure. **A)** The C_α RMSD (nm) of the protein was plotted as a function of time (ns). **B)** Ramachandran plot of the energy-minimized average structure from the final 3 ns of the 5-ns MD simulation. All residues are represented as black squares, except glycine, which is represented by black triangles.

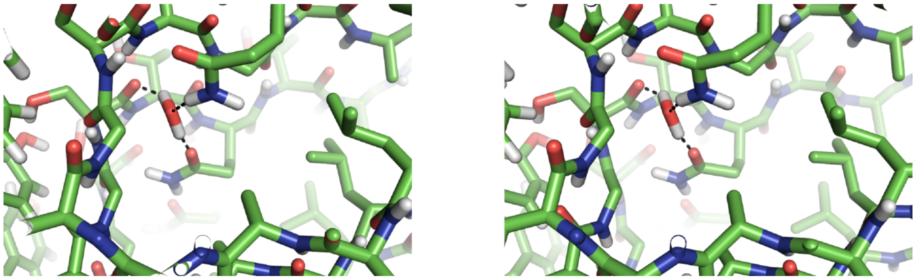

Figure S3 – Stereo view of water-bridged glutamine ladder. A glycine substitution typically interrupts the glutamine ladder every third loop in the model. This produces a void in the structure that is filled by a water molecule that bridges adjacent glutamine residues. Hydrogen bonds made by the water are shown by dashed lines.

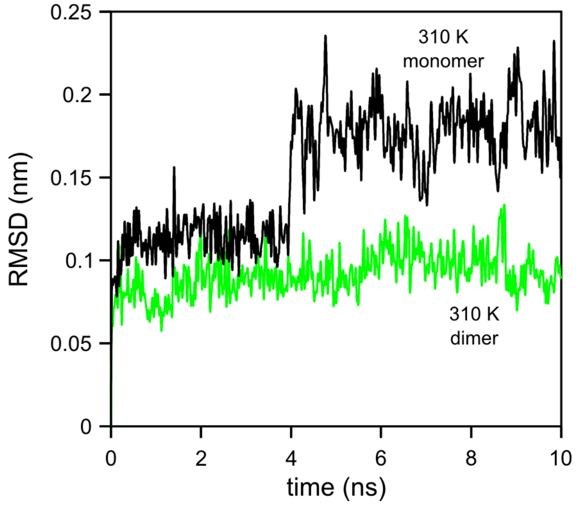

Figure S4 – RMSD plots of the *Pb*INP monomer and dimer simulated at 310 K for 10 ns. The C_α RMSD (nm) of both the monomer (black) and dimer (green) were plotted as a function of time (ns). The dimer remained stable throughout the entire trajectory, while the C terminus of the monomer began to unravel at the ca. 4-ns point.

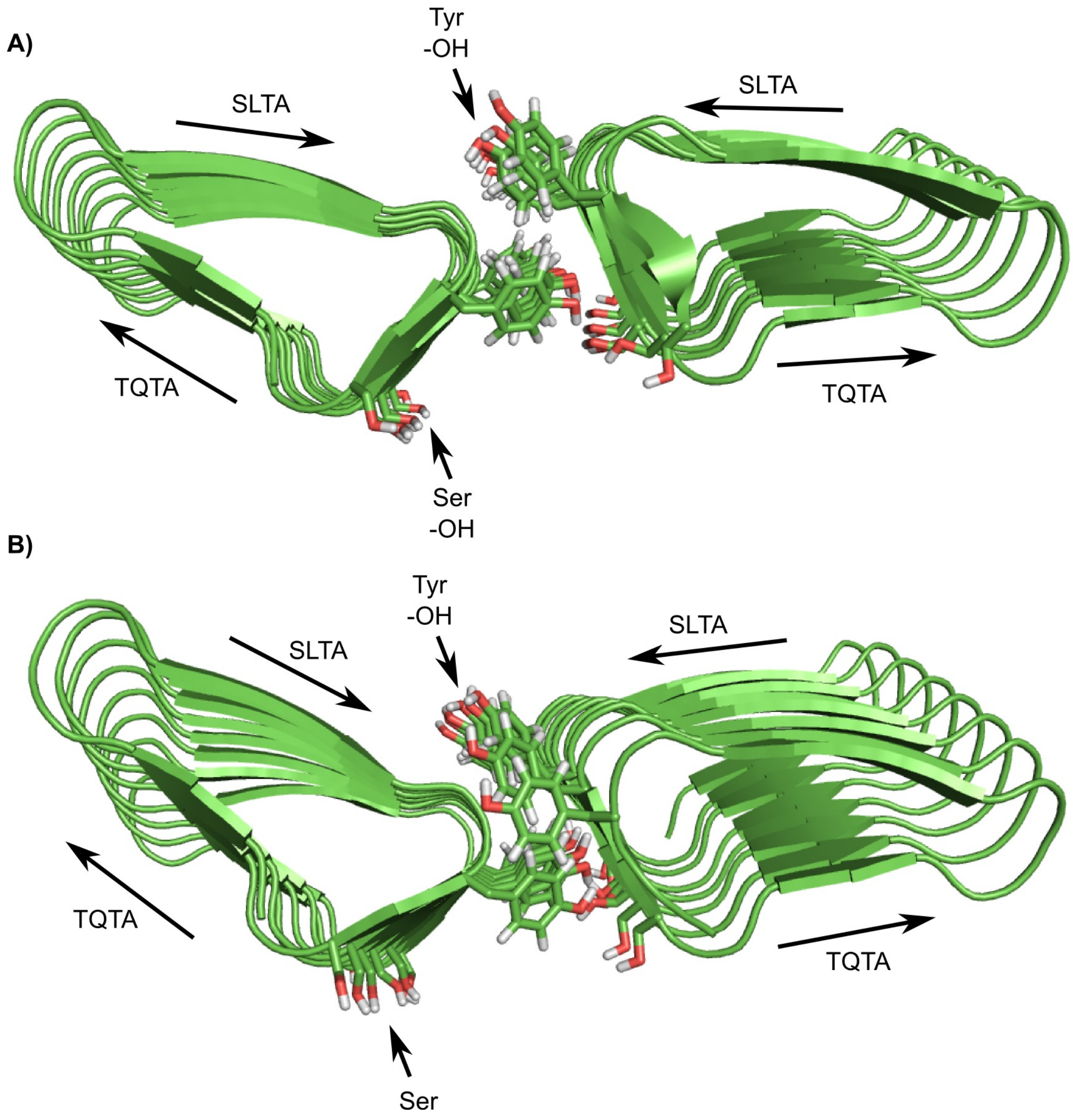

Figure S5 – Anti-parallel dimer of *Pb*INP. A) *Pb*INP aligned as an anti-parallel dimer prior to the start of a solvated 10-ns MD simulation. The unsatisfied rank of Ser and Tyr hydroxyl groups from adjacent chains are indicated. **B)** Energy-minimized average structure of *Pb*INP following the solvated 10-ns MD simulation. While the structure was stable, its flatness was spoiled due to a large twist that developed. When *Pb*INP is aligned as a parallel dimer, no twisting occurs, and this maintains the molecule's flatness, a characteristic of all ice-binding proteins.

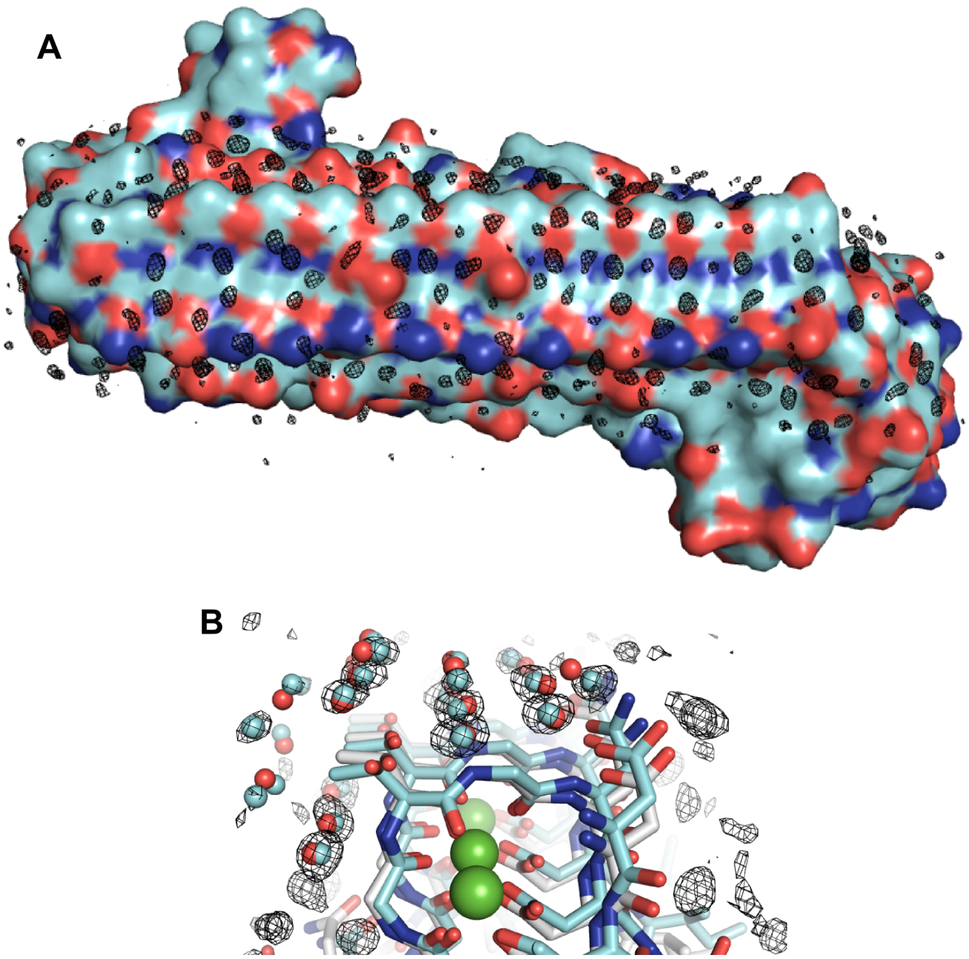

Figure S6 – Anchored clathrate waters on the IBS of *Mp*AFP_RIV. A) Chain B of *Mp*AFP_RIV is shown in surface mode, with carbons coloured light blue, oxygens red, and nitrogens dark blue. The water electron density (black mesh) is contoured at σ=6. **B)** The X-ray crystal structure of *Mp*AFP_RIV chain B (carbons coloured white) aligned to chain B from the MD simulation (carbons coloured light blue). Waters identified by crystallography are shown as red spheres, while waters built into the electron density (contoured at σ=6) are shown as light blue spheres. Ca^{2+} ions are represented as green spheres.
